# The Potential Bioactive Components of Nine TCM Prescriptions Against COVID-19 in Lung Cancer Were Explored Based on Network Pharmacology and Molecular Docking

**DOI:** 10.3389/fmed.2021.813119

**Published:** 2022-01-20

**Authors:** Lin Du, Yajie Xiao, Yijun Xu, Feng Chen, Xianghui Chu, Yuqi Cao, Xun Zhang

**Affiliations:** ^1^Department of Thoracic Surgery, Tianjin Chest Hospital, Tianjin, China; ^2^Department of Clinical Translational Medicine, YuceBio Technology Co., Ltd., Shenzhen, China

**Keywords:** COVID-19, Chinese medicine, network pharmacology, baicalein, molecular docking

## Abstract

**Objective:**

The purpose of this study was to screen active components and molecular targets of nine prescriptions recommended by the National Health Commission (NHC) of China by network pharmacology, and to explore the potential mechanism of the core active components against COVID-19 with molecular docking.

**Methods:**

Differentially expressed genes of lung adenocarcinoma (LUAD) screened by edgeR analysis were overlapped with immune-related genes in MMPORT and COVID-19-related genes in GeneCards. The overlapped genes were also COVID-19 immune-related genes in LUAD. TCMSP platform was used to identify active ingredients of the prescription, potential targets were identified by the UniProt database, and the cross genes with COVID-19 immune-related genes in LUAD were used to construct a Chinese Medicine-Logy-immune target network. Gene Ontology (GO) and Kyoto Encyclopedia of Genes and Genomes (KEGG) enrichment analyses were performed on the target genes of each prescription. Finally, the key active components were selected for molecular docking simulation with ACE2.

**Results:**

We obtained 15 overlapping immunization target genes from FPQXZ, HSYFZ, HSZFZ, and QFPDT, 16 overlapping immunization target genes from QYLFZ, SDYFZ, SRYFZ, and YDBFZ, and 17 overlapping immunization target genes from QYLXZ. ADRB2, FOS, HMOX1, ICAM1, IL6, JUN, NFKBIA, and STAT1 also had the highest-ranked therapeutic targets for 9 prescriptions, and their expressions were positively correlated with TME-related stromal score, immune score, and ESTIMATE score. Among 9 compounds with the highest frequency of occurrence in the 9 prescriptions, baicalein had the highest ACE2 binding affinity and can be well-combined into the active pocket of ACE2 It is stabilized by forming hydrogen bonds with ASN290 and ILE291 in ACE2 and hydrophobic interaction with PHE438, ILE291, and PRO415.

**Conclusion:**

The nine Chinese medicine prescriptions may play an anti-SARS-CoV-2 role *via* regulating viral transcription and immune function through multi-component, multi-target, and multi-pathway.

## Introduction

Coronavirus disease 2019 is a viral infection triggered by severe acute respiratory syndrome coronavirus 2 (SARS-CoV-2) (1). COVID-19 has spread rapidly, posing a serious threat to human health all over the world. Until August 11, 2021, there were nearly 205 million confirmed COVID-19 cases worldwide, resulting in more than 4.32 million deaths (https://www.worldometers.info/coronavirus/). It has been reported that patients with lung cancer are more likely to be infected with SARS-COV-2 and have a higher risk of death than normal ones due to impaired basic lung function and immunosuppression (2). The current challenge in treating patients with lung cancer is the balance between the risk of a potentially life-threatening infection with COVID-19 and the consequences of delayed treatment or non-treatment of lung cancer (3). Food and Drug Administration has approved antivirals, immune-modulators, nucleotide analogs, and convalescent plasma therapy for emergency treatment of COVID-19 (4). Many clinical studies have shown that the effectiveness of Chinese medicine interventional therapy for COVID-19 can reach higher than 90% (5). Recent clinical studies have combined both Chinese and Western medicine to treat COVID-19 with great success (6). The results of a meta-analysis demonstrated that integrated Traditional Chinese and Western medicine treatment for COVID-19 was more effective than applying conventional Western medicine treatment, with a better improvement of patients' clinical symptoms, chest CT and infection indicators (7). To date, there are more than 133 ongoing registered clinical studies of Chinese medicine/integrated Chinese and Western medicine (8). Although Chinese medicine has significant advantages in the treatment of COVID-19 and has strong clinical support, it is still considered as an alternative or complementary medicine mainly due to unspecific biochemical active ingredients of its prescriptions and unclear mechanism of action (9). Therefore, the problem that active ingredients and their mechanism of action should be explored.

The emergence of network pharmacology has provided great convenience for the study of pharmacological action and mechanisms of Chinese medicine. With network pharmacology, researchers can mine drug and disease targets from vast amounts of data to understand the mechanisms of action and regulatory pathways (10). In this study, we used online pharmacology to screen active ingredients and potential targets of nine prescriptions recommended by the National Health Commission (NHC) of China. Moreover, the potential mechanism of the anti-COVID-19 action of the core active ingredient through the molecular docking method was investigated. The current findings provide a reference for clinical treatment and mechanism study of Chinese medicine.

## Materials and Methods

### Data Gathering and Processing

**Figure 1:**
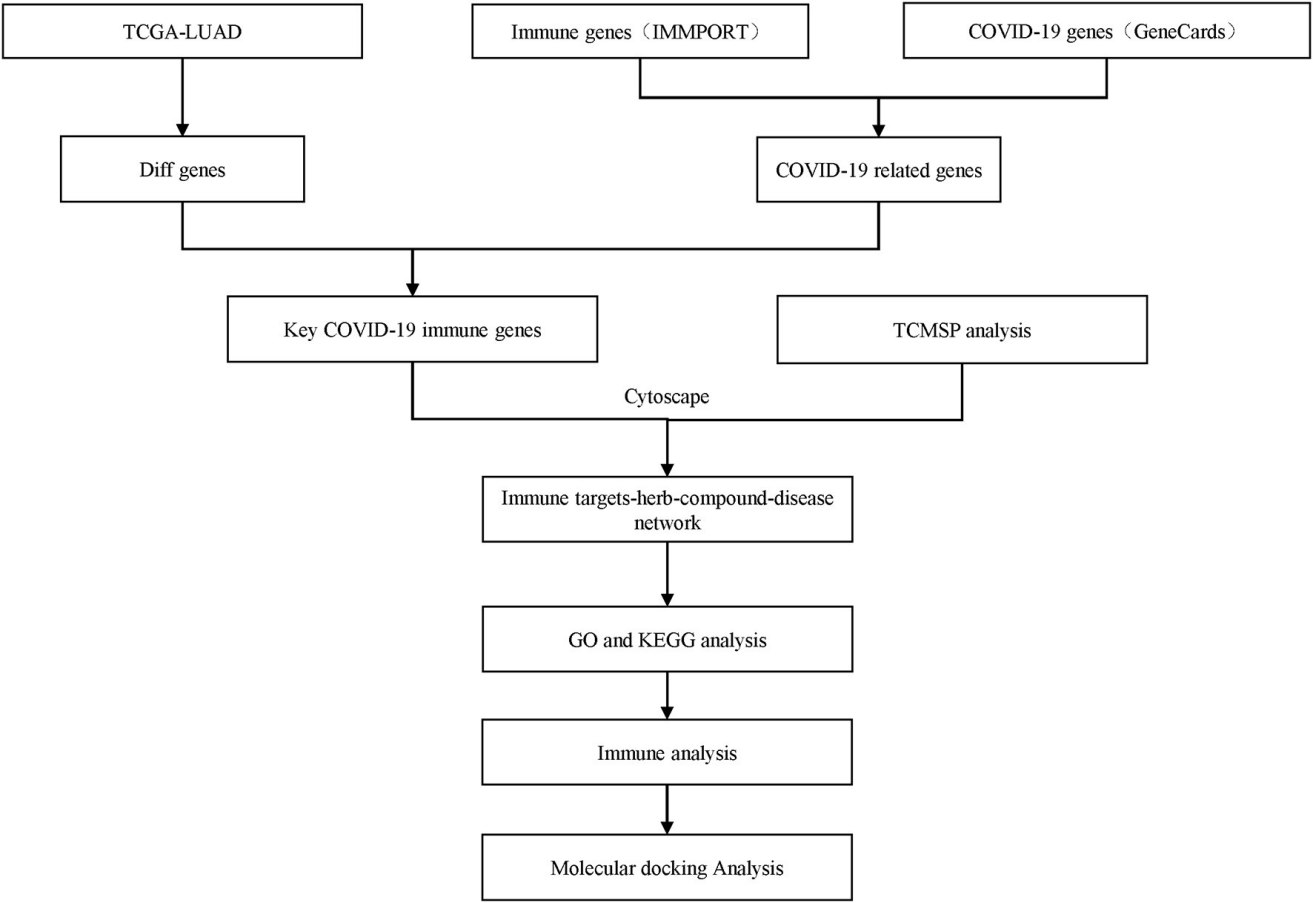
Workflow chart.

The IMMPORT (Division of Systems Medicine, Department of Pediatrics, Stanford University School of Medicine) (https://www.immport.org) database (11) was used to retrieve and collate 1,793 immune-related genes. A total of 1,566 COVID-19 related genes were acquired from Genecards (Department of Molecular Genetics, Weizmann Institute of Science) (https://www.genecards.org/) (12). The RNA sequencing data of TCGA-LUAD patients was obtained from the TCGA data portal (https://tcga-data.nci.nih.gov/tcga/). We downloaded the RNA-seq FPKM data set and further transformed the expression profile into transcripts per kilobase million (TPM). Differential expression of coding genes between tumor tissue and normal tissue was analyzed using edgeR (Institute of Molecular Life Sciences, University of Zurich) (13) (V3.26.8) to take the intersection of immune-related genes in MMPORT and COVID-19 related genes in GeneCards. The results of overlapping genes were shown by Venn Diagram (Informatics and Biocomputing Platform, Ontario Institute for Cancer Research) (14). The workflow chart is shown in Figure 1.

### Screening of Active Ingredients for COVID-19 Treatment and Prediction of Corresponding Targets

A total of 9 prescriptions of Chinese medicine were selected from “Novel Coronavirus Pneumonia Diagnosis and Treatment Scheme (Trial Edition 8)” issued by the National Health Commission of China, including Qing-Fei-Pai-Du-Tang (QFPDT), Shi-Re-Yun -Fei-Zheng (SDYFZ), Shi-Du-Yu-Fei-Zheng (SDYFZ), Han-Shi-Yu-Fei-Zheng (HSYFZ), Yi-Du-Bi-Fei-Zheng (YDBFZ), Han-Shi-Zu-Fei-Zheng (HSZFZ), Fei-Pi-Qi-Xu-Zheng (FPQXZ), Qi-Ying-Liang-Fan-Zheng (QYLFZ), and Qi-Yin-Liang-Xu-Zheng (QYLXZ). Bioactive ingredients of each prescription were searched through the Chinese Medicine Database and Analysis Platform (Center for Bioinformatics, College of Life Science, Northwest A&F University) (15) (TCMSP, https://tcmsp-e.com/). The most commonly used screening parameter for web-based pharmacological analysis was oral bioavailability (OB) (16), drug-likeness (DL) (17), and intestinal epithelial permeability (Caco-2) (18). In this method, the criteria of OB ≥ 30% and drug-likeness ≥ 0.18 were applied to screen bioactive components and their potential targets, and the target was annotated in UniProt (European Molecular Biology Laboratory, European Bioinformatics Institute (EMBL-EBI), Wellcome Trust Genome Campus) (https://www.uniprot.org/). The intersected genes of COVID-19 target genes and prescription target gene sets using The Venn Diagram package in the R software.

### Construction of Chinese Medicine-Constituent-Immune Target

To construct the Chinese medicine herb-constituent-immune target network, CytoScape (Institute for Systems Biology) (V3.7.2) (19) was used in order to reflect the complex relationship among active Chinese medicine, compounds, and filtrated targets. Topology analysis of networks was carried out according to the values of degree centrality, betweenness centrality, and closeness centrality. The protein-protein interaction data set comes from the stringdb database (https://cn.string-db.org/) (Department of Molecular Life Sciences and Swiss Institute of Bioinformatics, University of Zurich) (20).

### Gene Ontology (GO) and KEGG Pathway Enrichment Analysis

To conduct GO and KEGG enrichment analyses, the ClusterProfiler (Institute of Life and Health Engineering, Key Laboratory of Functional Protein Research of Guangdong Higher Education Institutes, Jinan University) database was used by importing the list of intersection target gene names and setting the species as “hsa” for customized analysis with a filter *P*-value of < 0.05.

### Simulated Molecular Docking

The active ingredient with the highest content in nine kinds of Chinese medicine was selected, and the chemical structure of the active ingredient and ACE2 was downloaded from PubChem (National Center for Biotechnology Information, National Library of Medicine, National Institutes of Health, Department of Health and Human Services). AutoDock Vina software (Department of Molecular Biology, The Scripps Research Institute) (21) was used to simulate molecular docking. AutoDockTools processed the ACE2 protein, added polar hydrogen, calculated the Gasteiger charge, and set all ligand rotatable bonds. The targets and drugs were prepared and molecular docking performed inside a grid box (40 Å × 40 Å × 40 Å). A lamarckian genetic algorithm was applied to calculate protein docking with the ligand. All docking was run with default settings. The exhaustiveness level was set to 8 and the output maximum was set to 10.

## Results

### Collection of Active Ingredients and Target Gene Screening for Prescribing Treatments for COVID-19

**Figure 2:**
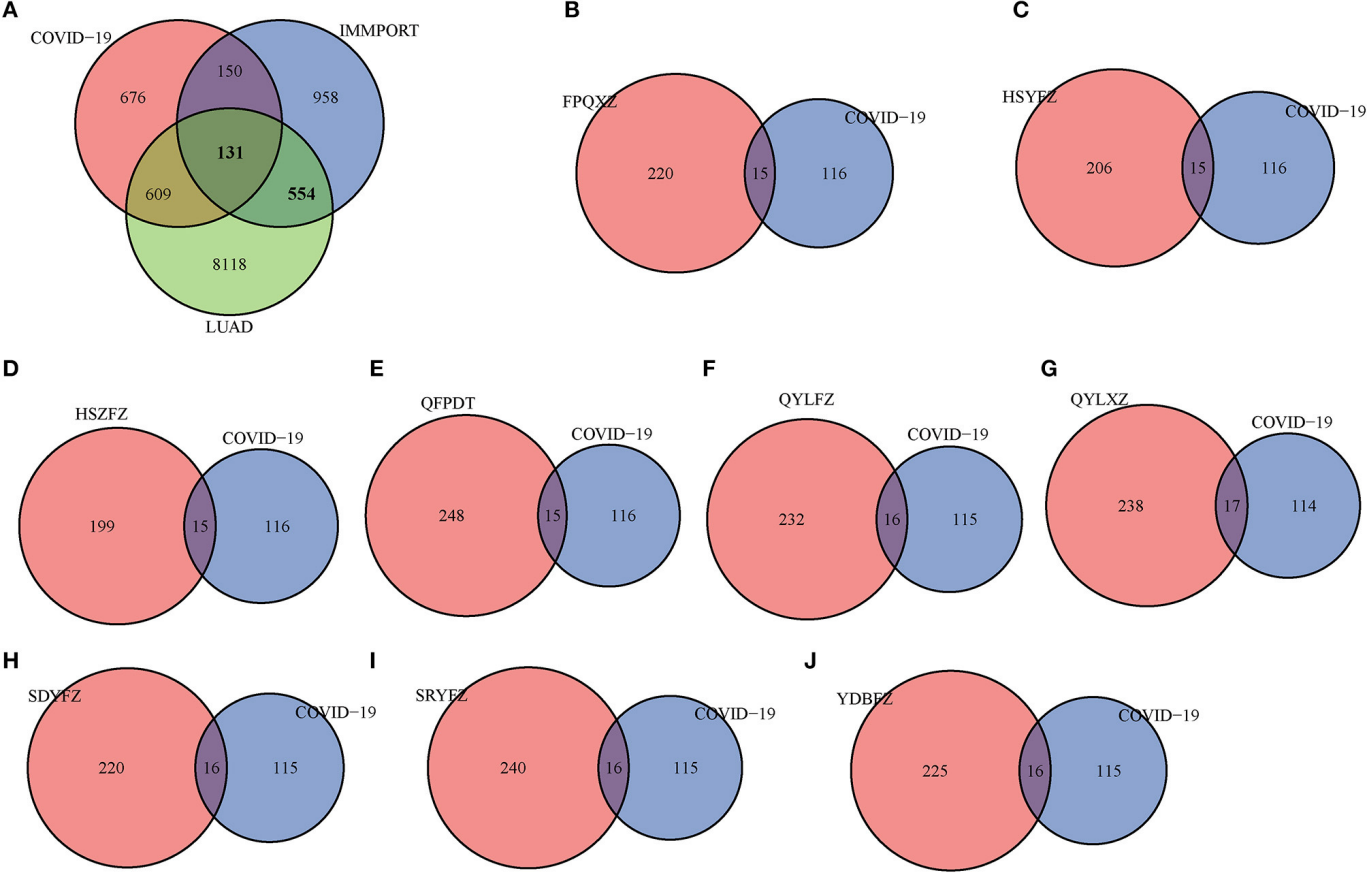
Active ingredients and targets prescribed for COVID-19 treatment. **(A)** Intersection analysis of differentially expressed genes, immune-related genes, and COVID-19 related genes in CLUAD. The Venn diagram of FPQXZ **(B)**, HSYFZ **(C)**, HSZFZ **(D)**, QFPDT **(E)**, QYLFZ **(F)**, QYLXZ **(G)**, SDYFZ **(H)**, SRYFZ **(I)**, and YDBFZ **(J)** with COVID-19 immune-related genes in LUAD.

A total of 9,412 differentially expressed genes were screened from TCGA-LUAD by performing differential analysis. One hundred and thirty-one genes were determined as COVID-19 immune-related and LUAD-related (Figure 2A). According to OB value and DL index, the active ingredients of nine prescriptions were obtained from TCMSP. Then the target genes of each prescription were predicted to take the intersection with COVID-19 immune-related genes in LUAD. In this study, 15 immune target genes were obtained from FPQXZ, HSYFZ, HSZFZ, and QFPDT, 16 immune target genes were obtained from QYLFZ, SDYFZ, SRYFZ, and YDBFZ, and 17 were obtained from QYLXZ (Figures 2B–J). We used WebGestaltR to analyze the function of these gene immune target genes. We can observe that they are mainly related to cytokine receptor interaction, JAK-STAT signaling pathway, and MAPK signaling pathway, such as Supplementary Figure 1A. In addition, they are also related to blood vessel morphogenesis, positive regulation of MAPK cascade Regulation of signaling receiver activity is related to biological processes (Supplementary Figure 1B). Cell composition analysis shows that these genes are mainly enriched in the side of the membrane, receiver complex, external side of the plasma membrane, and other components (Supplementary Figure 1C). They are also enriched in receiver regulator activity, receiver live activity Cytokine receptor binding, and other molecular functions (Supplementary Figure 1D). These results prove the relationship between these genes and the immune process.

### Construction of Chinese Medicine-Constituent-Immune Target Network

**Figure 3:**
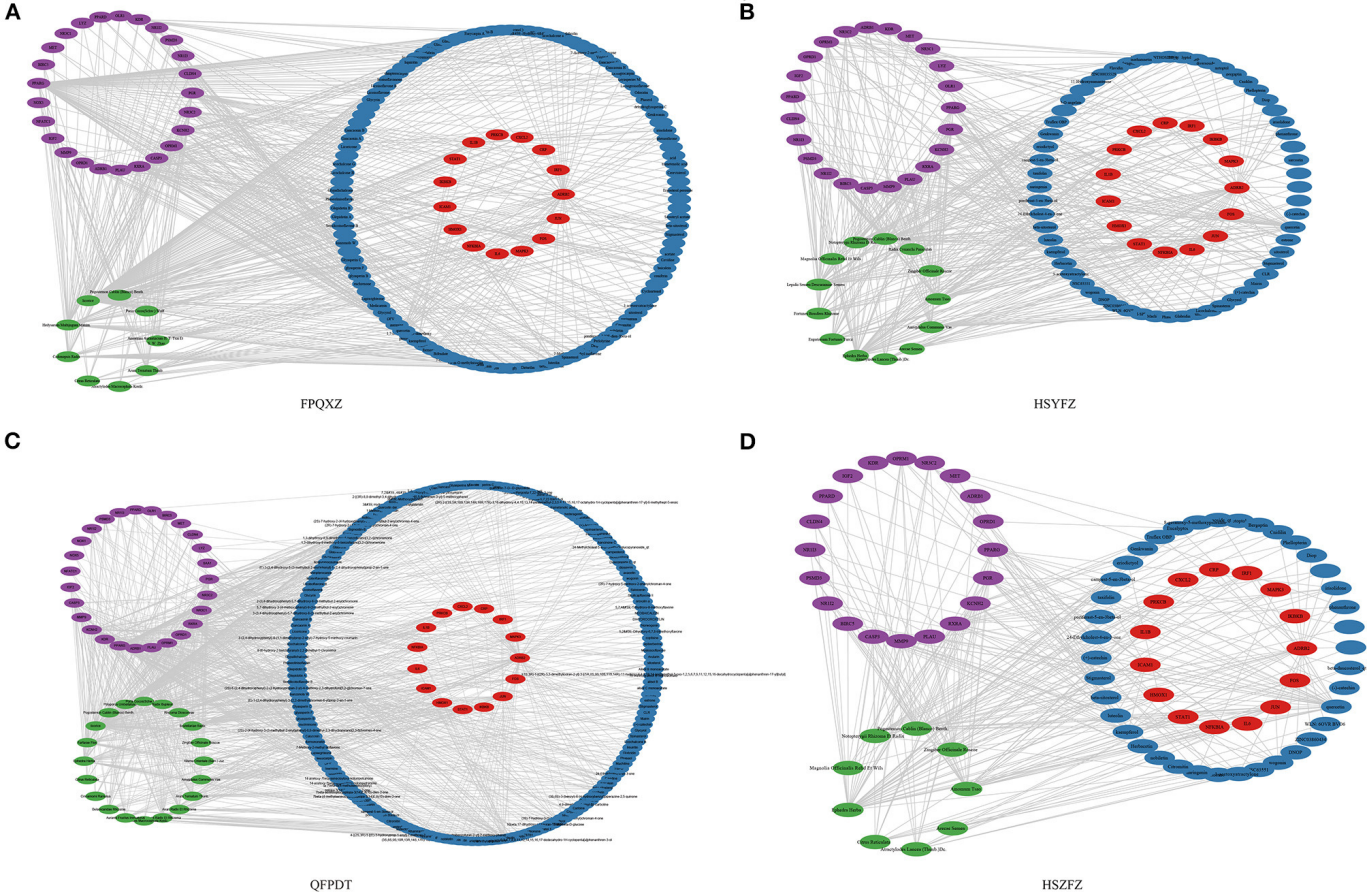
The Chinese medicine herb- constituent -immune target network. **(A)** FPQXZ's Chinese medicine herb—constituent -immune target regulatory network. **(B)** HSYFZ's Chinese medicine herb—constituent -immune target network. **(C)** QFPDT's Chinese medicine herb—constituent -immune target network. **(D)** Chinese medicine herb—constituent -immune target interaction network of HSZFZ.

The interaction network of the Chinese medicine-constituent-immune target network was constructed by introducing each Chinese medicine prescription, its active ingredient, and predicted target into Cytoscape. As shown in Figure 3 and Supplementary Figure 2, the nodes of the Chinese medicine—constituent -immune target network of each prescription could be observed. Among them, green, blue, red, and purple circles represented Chinese medicine prescription, compounds, immune target genes, and non-immune target genes of the prescription, respectively. We also analyzed the degree distribution and eigenvector centrality of each network. It can be observed that the moderate distribution of these networks presents a dark rate form, which is consistent with the characteristics of biological networks. The eigenvector centrality shows a similar situation, as shown in Supplementary Figure 3, In In each Chinese medicine prescription network, one compound acted on a multitarget, and different compounds could also simultaneously interact with an immune target. Moreover, the top 10 key compounds of the top 10 core targets in FPQXZ were identified according to the degree value as shown in Tables 1, 2. Table 3 showed predicted targets of the top 10 active ingredients, with their corresponding genes in FPQXZ.

**Table 1:** The top 10 key compounds in FPQXZ.

**Compound**	**Degree**	**Betweenness centrality**
Quercetin	29	0.01015208
Kaempferol	13	0.00336682
Luteolin	11	0.00193848
Stigmasterol	9	0.0006946
Beta-sitosterol	8	0.00041647
Baicalein	7	0.0016664
Irisolidone	7	0.00038325
Cavidine	7	0.00034745
7-O-methylisomucronulatol	7	0.00034529
Shinpterocarpin	7	0.00030962

**Table 2:** The top 10 core targets in FPQXZ.

**Gene**	**Degree**	**Betweenness centrality**
ADRB2	50	0.07531967
JUN	14	0.00682336
ICAM1	7	0.00088071
HMOX1	7	0.00088071
FOS	6	0.00214445
NFKBIA	6	0.00073893
IL6	6	0.00073893
IL1B	5	0.0002301
STAT1	5	8.54E-05
IRF1	4	4.83E-05

**Table 3:** Predicted targets of the top 10 active ingredients, with their corresponding genes in FPQXZ.

**MolId**	**Mol name**	**Target**	**Symbol**	**Herb**
MOL000098	Quercetin	Beta-2 adrenergic receptor	ADRB2	Hedysarum Multijugum Maxim.
MOL000098	Quercetin	Beta-2 adrenergic receptor	ADRB2	Licorice
MOL000098	Quercetin	Beta-2 adrenergic receptor	ADRB2	Pogostemon Cablin (Blanco) Benth.
MOL000449	Stigmasterol	Beta-2 adrenergic receptor	ADRB2	Amomum Aurantiacum H. T. Tsai Et S. W. Zhao
MOL000449	Stigmasterol	Beta-2 adrenergic receptor	ADRB2	Arum Ternatum Thunb.
MOL000449	Stigmasterol	Beta-2 adrenergic receptor	ADRB2	Codonopsis Radix
MOL000358	Beta-sitosterol	Beta-2 adrenergic receptor	ADRB2	Amomum Aurantiacum H. T. Tsai Et S. W. Zhao
MOL000358	Beta-sitosterol	Beta-2 adrenergic receptor	ADRB2	Arum Ternatum Thunb.
MOL005916	Irisolidone	Beta-2 adrenergic receptor	ADRB2	Pogostemon Cablin (Blanco) Benth.
MOL002670	Cavidine	Beta-2 adrenergic receptor	ADRB2	Arum Ternatum Thunb.
MOL000378	7-O-methylisomucronulatol	Beta-2 adrenergic receptor	ADRB2	Hedysarum Multijugum Maxim.
MOL004891	Shinpterocarpin	Beta-2 adrenergic receptor	ADRB2	Licorice
MOL000098	Quercetin	Transcription factor AP-1	JUN	Hedysarum Multijugum Maxim.
MOL000098	Quercetin	Transcription factor AP-1	JUN	Licorice
MOL000098	Quercetin	Transcription factor AP-1	JUN	Pogostemon Cablin (Blanco) Benth.
MOL000422	Kaempferol	Transcription factor AP-1	JUN	Hedysarum Multijugum Maxim.
MOL000422	Kaempferol	Transcription factor AP-1	JUN	Licorice
MOL000006	Luteolin	Transcription factor AP-1	JUN	Codonopsis Radix
MOL000358	Beta-sitosterol	Transcription factor AP-1	JUN	Amomum Aurantiacum H. T. Tsai Et S. W. Zhao
MOL000358	Beta-sitosterol	Transcription factor AP-1	JUN	Arum Ternatum Thunb.
MOL005916	Irisolidone	Transcription factor AP-1	JUN	Pogostemon Cablin (Blanco) Benth.
MOL000098	Quercetin	Intercellular adhesion molecule 1	ICAM1	Hedysarum Multijugum Maxim.
MOL000098	Quercetin	Intercellular adhesion molecule 1	ICAM1	Licorice
MOL000098	Quercetin	Intercellular adhesion molecule 1	ICAM1	Pogostemon Cablin (Blanco) Benth.
MOL000422	Kaempferol	Intercellular adhesion molecule 1	ICAM1	Hedysarum Multijugum Maxim.
MOL000422	Kaempferol	Intercellular adhesion molecule 1	ICAM1	Licorice
MOL000006	Luteolin	Intercellular adhesion molecule 1	ICAM1	Codonopsis Radix
MOL000098	Quercetin	Heme oxygenase 1	HMOX1	Hedysarum Multijugum Maxim.
MOL000098	Quercetin	Heme oxygenase 1	HMOX1	Licorice
MOL000098	Quercetin	Heme oxygenase 1	HMOX1	Pogostemon Cablin (Blanco) Benth.
MOL000422	Kaempferol	Heme oxygenase 1	HMOX1	Hedysarum Multijugum Maxim.
MOL000422	Kaempferol	Heme oxygenase 1	HMOX1	Licorice
MOL000006	Luteolin	Heme oxygenase 1	HMOX1	Codonopsis Radix
MOL000098	Quercetin	Proto-oncogene c-Fos	FOS	Hedysarum Multijugum Maxim.
MOL000098	Quercetin	Proto-oncogene c-Fos	FOS	Licorice
MOL000098	Quercetin	Proto-oncogene c-Fos	FOS	Pogostemon Cablin (Blanco) Benth.
MOL002714	Baicalein	Proto-oncogene c-Fos	FOS	Arum Ternatum Thunb.
MOL000098	Quercetin	NF-kappa-B inhibitor alpha	NFKBIA	Hedysarum Multijugum Maxim.
MOL000098	Quercetin	NF-kappa-B inhibitor alpha	NFKBIA	Licorice
MOL000098	Quercetin	NF-kappa-B inhibitor alpha	NFKBIA	Pogostemon Cablin (Blanco) Benth.
MOL000006	Luteolin	NF-kappa-B inhibitor alpha	NFKBIA	Codonopsis Radix
MOL000098	Quercetin	Interleukin-6	IL6	Hedysarum Multijugum Maxim.
MOL000098	Quercetin	Interleukin-6	IL6	Licorice
MOL000098	Quercetin	Interleukin-6	IL6	Pogostemon Cablin (Blanco) Benth.
MOL000006	Luteolin	Interleukin-6	IL6	Codonopsis Radix
MOL000098	Quercetin	Interleukin-1 beta	IL1B	Hedysarum Multijugum Maxim.
MOL000098	Quercetin	Interleukin-1 beta	IL1B	Licorice
MOL000098	Quercetin	Interleukin-1 beta	IL1B	Pogostemon Cablin (Blanco) Benth.
MOL005916	Irisolidone	Interleukin-1 beta	IL1B	Pogostemon Cablin (Blanco) Benth.
MOL000098	Quercetin	Signal transducer and activator of transcription 1-alpha/beta	STAT1	Hedysarum Multijugum Maxim.
MOL000098	Quercetin	Signal transducer and activator of transcription 1-alpha/beta	STAT1	Licorice
MOL000098	Quercetin	Signal transducer and activator of transcription 1-alpha/beta	STAT1	Pogostemon Cablin (Blanco) Benth.
MOL000422	Kaempferol	Signal transducer and activator of transcription 1-alpha/beta	STAT1	Hedysarum Multijugum Maxim.
MOL000422	Kaempferol	Signal transducer and activator of transcription 1-alpha/beta	STAT1	Licorice
MOL000098	Quercetin	Interferon regulatory factor 1	IRF1	Hedysarum Multijugum Maxim.
MOL000098	Quercetin	Interferon regulatory factor 1	IRF1	Licorice
MOL000098	Quercetin	Interferon regulatory factor 1	IRF1	Pogostemon Cablin (Blanco) Benth.

### Analysis of GO Function and KEGG Enrichment of Related Targets

**Figure 4:**
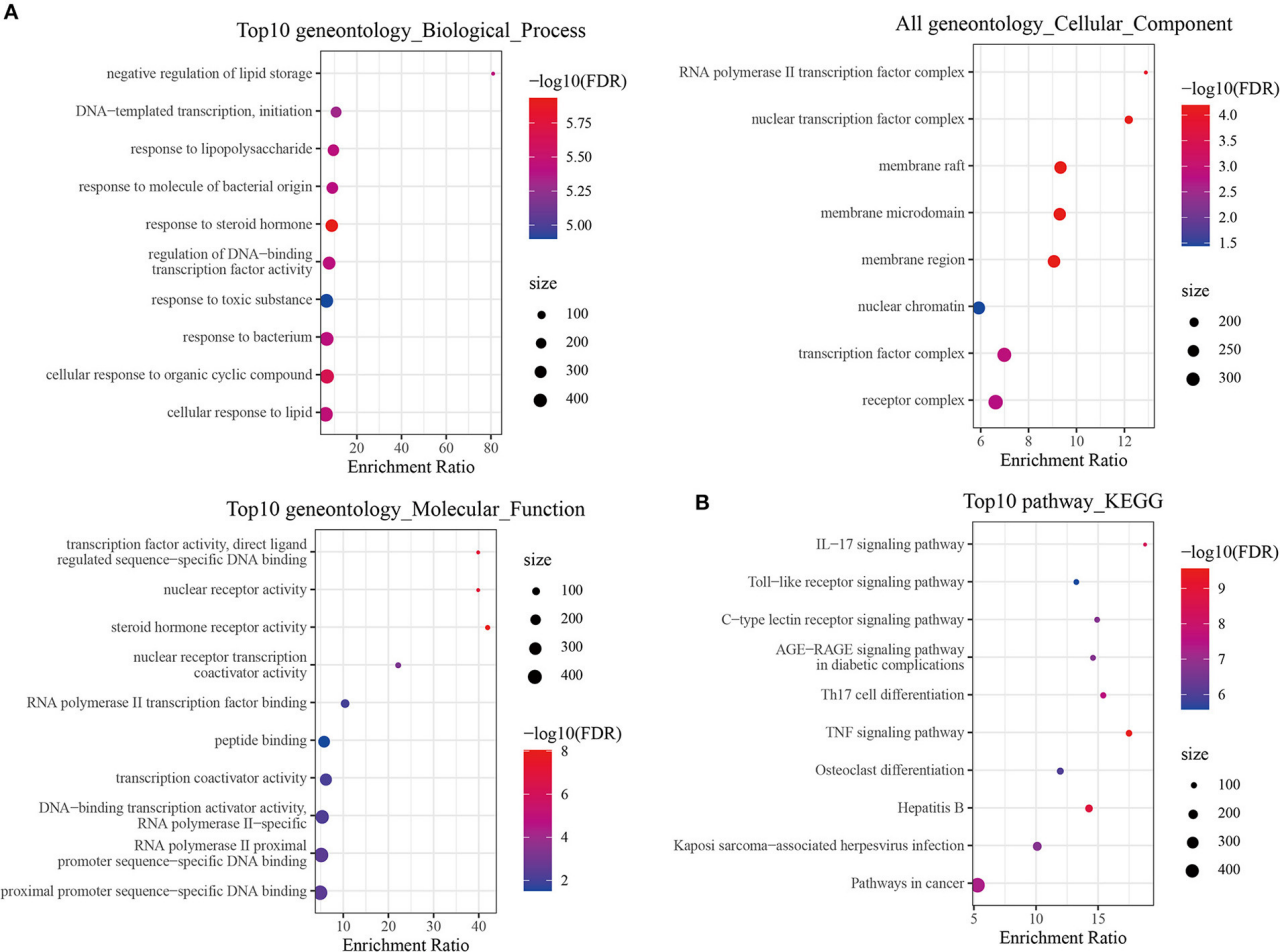
GO and KEGG analysis of FPQXZ target genes. **(A)** GO terms of FPQXZ target genes. The top 10 GO functional categories were selected. **(B)** KEGG bubble diagram showed the top 10 pathways of FPQXZ target genes.

Gene ontology (GO) and KEGG analyses were performed on the targets of each prescription to obtain enriched ontology clusters. A total of 309 GO terms including 284 biological processes (BPs), eight cellular components (CCs), and 17 molecular functions (MFs) were found in the GO functional annotation of FPQXZ target genes (Figure 4A). For BPs, the targets were mainly involved in transcriptional regulation, response to hormones, and harmful factors. CCs terms demonstrated that the targets were associated with transcription factor complex and membrane raft. From MFs analysis, it could be found that the targets were mainly associated with nuclear receptor activity, steroid hormone receptor activity, peptide binding, etc. The enrichment analysis of KEGG signal pathways included 87 pathways. From the visual results of the first 10 pathways, the target gene of the main signaling pathways of prescription was concentrated in pathways in the IL−17 signaling pathway, Th17 cell differentiation, TNF signaling pathway, Hepatitis B, etc. (Figure 4B). The GO function and KEGG enrichment results of the target genes of the other eight Chinese medicine prescriptions were displayed in Supplementary Figure 4.

### Correlation Between Tumor Environment (TME) and Immune Target Gene of Chinese Medicine Prescription

**Figure 5:**
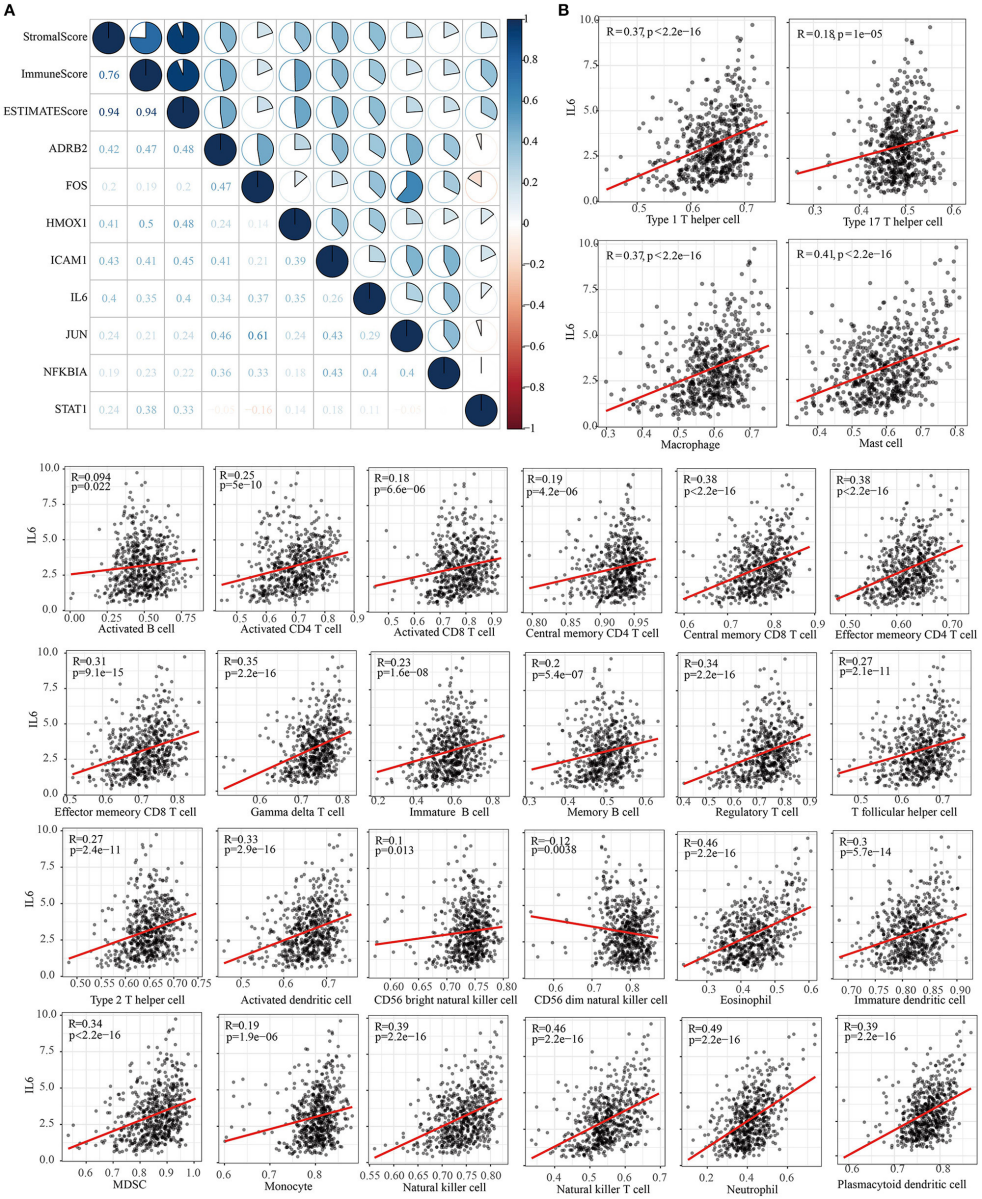
Correlation analysis between immune target genes and TME in Chinese medicine prescription. **(A)** Correlation between immune target genes and stromal score, immune score, and ESTIMATE score in Chinese medicine prescription. **(B)** Pearson correlation analysis between IL6 expression level and ssGSEA score of 28 immune cells.

Comparison analysis showed that eight (ADRB2, FOS, HMOX1, ICAM1, IL6, JUN, NFKBIA, and STAT1) out of the top 10 immune target genes of each prescription were therapeutic targets of 9 kinds of prescription (Table 4). Eight immune target genes were significantly differentially expressed in cancer and adjacent tumors. Except for STAT1, the other seven genes were low expressed in tumor samples (Supplementary Figure 5A). Stromal score, immune score, and ESTIMATE score of LUAD in TCGA were obtained through ESTIMATE, and correlation analysis was conducted between these TME-related scores and 8 immune target genes. Three TME-related scores were found to be positively correlated with 8 immune target genes (Figure 5A). According to single-gene GSEA (ssGSEA), the infiltration score of 28 types of immune cells was assessed, including activated B cell, activated CD4 T cell, activated CD8 T cell, central memory CD4 T cell, central memory CD8 T cell, effector memory CD4 T cell, effector memory CD8 T cell, gamma delta T cell, immature B cell, memory B cell, regulatory T cell, follicular helper T cell, type 1 T helper cell, type 17 T helper cell, type 2 T helper cell, activated dendritic cell, CD56 bright natural killer cell, CD56 dim natural killer cell, eosinophil, immature dendritic cell, macrophage, mast cell, MDSC, monocyte, natural killer cell, natural killer T cell, neutrophil, plasmacytoid dendritic cell. The Pearson correlation analysis between IL6 and 28 kinds of immune cells showed that the expression of IL6 was negatively correlated with the scores of CD56 dim natural killer cell, and positively correlated with the scores of the other 27 kinds of cells (Figure 5B).

**Table 4:** The common immune target genes of each prescription were counted.

**Gene**	**Freq**
ADRB2	9
FOS	9
HMOX1	9
ICAM1	9
IL6	9
JUN	9
NFKBIA	9
STAT1	9
IL1B	7
PRKCB	7
IKBKB	3
IRF1	1

### Molecular Docking

**Figure 6:**
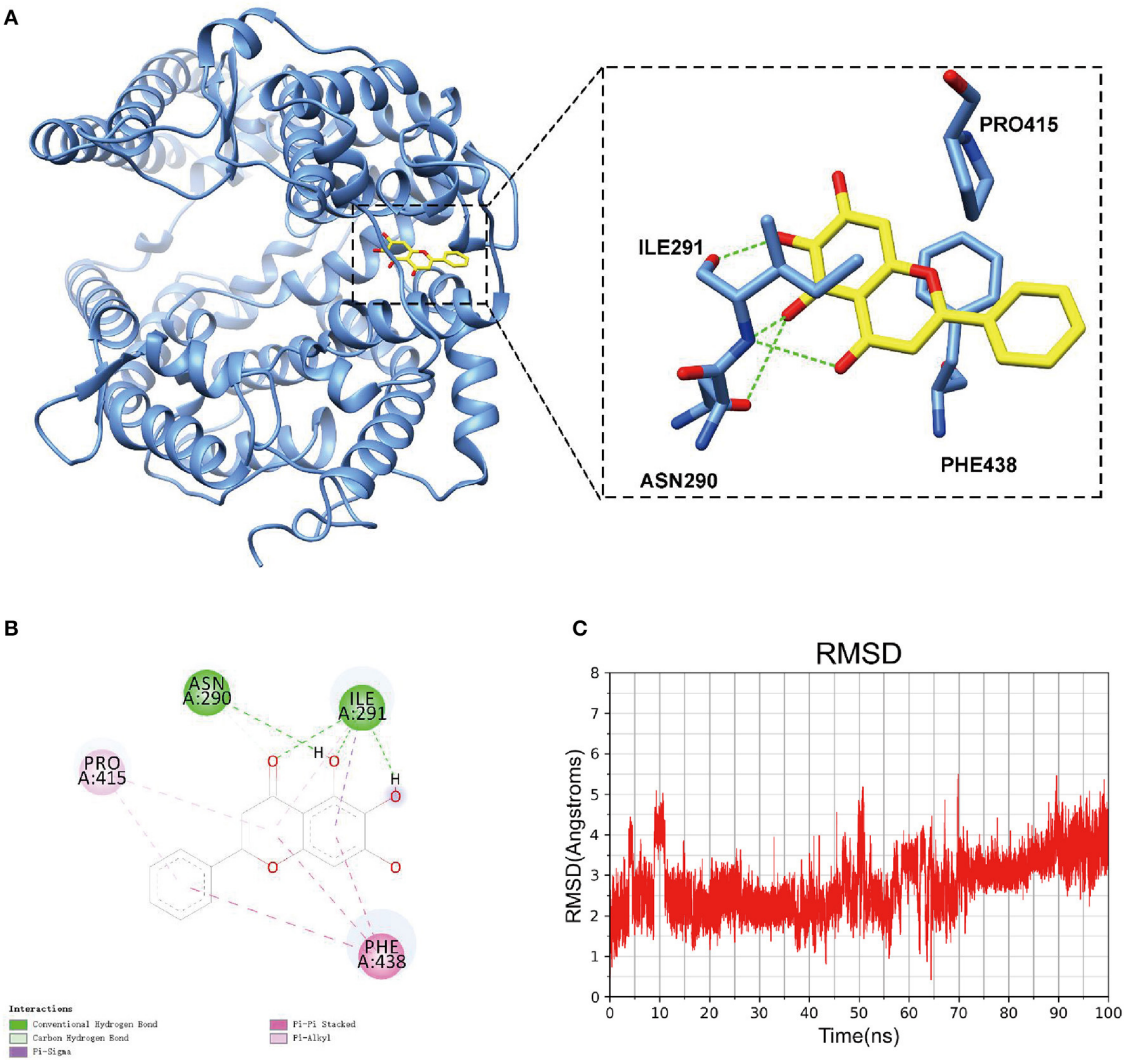
Molecular docking patterns of baicalein and ACE2. **(A)** The pose of baicalein within the binding site of ACE2. The yellow confirmation was the baicalein, and the green dotted line was the hydrogen bond. **(B)** 2D-interaction diagram of ACE2 -baicalein interaction. **(C)** RMSD of baicalein combined with ACE2 for 100 ns.

Among the top 10 compounds in each prescription, we selected the compounds that occur more than or equal to five times in nine prescriptions, and these included beta-sitosterol, kaempferol, luteolin, quercetin, stigmasterol, shinpterocarpin, wogonin, baicalein, and irisolidone (Table 5). The docking results of the nine compounds with ACE2 were shown in Table 6. The binding free energy of each compound to ACE2 was lower than 0, indicating that all the nine compounds could well-bind to ACE2, and baicalein had the highest binding affinity to ACE2 among all the nine compounds. So the molecular docking pattern between baicalein and ACE2 was simulated here. It was found that baicalein could well-bind to the active pockets of ACE2, and was stabilized through forming hydrogen bonds with ASN290 and ILE291 in ACE2 and hydrophobic interactions with PHE438, ILE291, and PRO415 (Figures 6A,B). The 100 ns simulation of baicalein combined with ACE2 showed the conformational change of baicalein in the binding pocket of ACE2, and that the root mean square deviation (RMSD) of each moment was almost the same (Figure 6C). These findings indicated that baicalein may bind to ACE2, thereby inhibiting the host-virus protein interactions in which they were involved.

**Table 5:** The frequency of occurrence of different compounds in nine prescriptions.

**Compound**	**Freq**
Beta-sitosterol	9
Kaempferol	9
Luteolin	9
Quercetin	9
Stigmasterol	9
Shinpterocarpin	6
Wogonin	6
Baicalein	5
Irisolidone	5
Sitosterol	4
Cavidine	3
7-Methoxy-2-methyl isoflavone	2
7-O-methylisomucronulatol	2
Estrone	2
l-SPD	2
Naringenin	2
1-Methoxyphaseollidin	1
Arachidonic acid	1
Cryptotanshinone	1
Eucalyptol	1
Nobiletin	1
Tanshinone iia	1

**Table 6:** Binding free energy of nine compounds to ACE2.

**Compounds**	**Score (kcal/mol)**	**Hydrogen-bond**	**Hydrophobic-bond**
Baicalein	−9.6	ASN290, ILE291	PHE438, ILE291, PRO415
Stigmasterol	−9.3	GLU406	PHE438, LEU370, ALA413, ILE291, LYS441
Beta-sitosterol	−9.3	GLU406	LEU370, MET366, ALA413
			PRO415, ILE291, PHE438, LYS441
Luteolin	−9.1	ILE291	PHE438, ILE291, THR434, PRO415
Kaempferol	−8.8	ASN290, ILE291, HIS540	PHE438, ALA413, THR434, PRO415
Wogonin	−8.5	ASP431, THR434	PHE438, PRO415, ILE291
Irisolidone	−8.3	LYS423	PHE420, PRO397, ILE273, MET348
Shinpterocarpin	−8.2	SER44, ALA348	ASP350, PHE40
Quercetin	−7.7	ALA413, ASP367, THR434	PHE438, MET366

## Discussion

The interaction of COVID-19 virus spike protein with host angiotensin-converting enzyme 2 (ACE2) receptor is the primary mechanism for SARS-CoV-2 entry into host cells (22). Therefore, a strategy that interferes with such an interaction may be an effective strategy for treating SARS-CoV-2. There is already a wealth of research data and findings in this area. Suresh Gangadevi et al. (23) showed that blocking the interaction between ACE2 receptor and S1-RBD *in vitro* could serve as a lead compound against COVID-19. Withanone in *Withania Somnifera* effectively inhibited the interaction between SARS-CoV-2 RBD and host ACE2 in a dose-dependent manner, and this can be used as a potential antiviral drug (24). A recent study applied multidisciplinary approaches to show that 12 ACE2 binders and 6 of the RBD binders competed with the RBD-ACE2 interaction, which may be explored as inhibitors to SARS-COV-2 (25). Some Chinese medicine prescriptions have also been clinically proven to be effective in treating COVID-19, however, due to their multi-component and multi-target characteristics, the active ingredients and mechanisms remain unknown.

This study applied network pharmacology to explore the active ingredients and therapeutic targets of nine approved Chinese medicine prescriptions, and combined with simulated molecular docking to analyze the important components with a high binding ability to COVID-19 targets. We screened eight common immune target genes from nine Chinese medicine prescriptions. JUN, NFKBIA, and ICAM1 have been reported as key genes related to COVID-19 (26). ADRB2, FOS, and IL-6 were found to be associated with COVID-19 inflammation (27–29). Dysregulation of HMOX1 was relevant to ARS-CoV-2 and cancer (30). A study of Toshifumi Matsuyama indicated that enhanced STAT1 activity could be used in the treatment of COVID-19 (31). In addition, we also found that ADRB2 and STAT1 were significantly correlated with the prognosis of patients with lung cancer (Supplementary Figure 5B). ADRB2 was a protective factor, while STAT1 was a risk factor. ADRB2 was closely related to a variety of diseases, such as ADRB2 signaling by inhibiting HIF1 α Autophagy degradation to promote HCC progression and sorafenib resistance (32). ADRB2 hypermethylation induced β 2AR down regulation inhibits PI3K / Akt, resulting in cardiac dysfunction (33). STAT1 involves M1 macrophage polarization, which may affect osteolysis and bone remodeling of extrapulmonary tuberculosis (34). STAT1 activation induces PRMT1 expression and regulates the remodeling of primary human lung fibroblasts (35). In addition, we also analyzed the expression relationship between these eight genes and ACE2. Generally speaking, the correlation between these genes and ACE2 is weak, suggesting that these eight genes may not directly co-express with ACE2 and participate in the immune pathway (Supplementary Figure 5C). Here, we found that they were positively correlated with TME, and the inhibition of the nine Chinese medicine prescriptions on COVID-19 may be partially achieved *via* acting on these targets.

We also listed the top 10 compounds in each prescription and identified at least nine compounds with high content in the five prescriptions. Through literature review, we learned that hydrogen bonding, hydrophobic, and van der Waals force interactions between Beta-Sitosterol and ACE2 (36). Kaempferol only formed a single hydrogen bond with ACE2 with low affinity but showed a high affinity with Akt1 (37, 38). Luteolin, quercetin can form strong hydrogen bonds with polar amino acid residues R273, D269, and N149 in ACE2 pocket, and weak hydrogen bonds with hydroxyl groups of Y127. At the same time, the top dihydroxyl group can form a double hydrogen bond with the -Co group on the N149 skeleton, with a free energy of binding of −7.92 kcal/mol (37), which was close to our estimated binding free energy of −7.7 kcal/mol. In addition, a study has calculated that the free energy of the combination of stigmasterol and ACE2 was −8.3 kcal/mol (39), and this was also consistent with our calculation. Tao et al. (40) reported that baicalein regulated multiple signaling pathways through ACE2. However, the conformation and stability of the two combinations are still unclear. Here, our results indicated that baicalein was an active component with the highest free binding energy to ACE2 among the nine compounds, and can be embedded in the active pocket of ACE2 to form a stable conformation through the formation of hydrogen bonds and hydrophobic interactions.

Our results suggested that baicalein may be the core ingredient in several Chinese medicine prescriptions for the effective treatment of COVID-19. This study may provide a theoretical basis for the development of anti-COVID-19 drugs. Even so, network pharmacology only analyzed the main active components and targets of drugs, its predicted targets and specific regulatory mechanisms need further experimental verification and exploration.

## Data Availability Statement

Publicly available datasets were analyzed in this study. This data can be found at: the IMMPORT (https://www.immport.org) database was used to retrieve and collate 1,793 immune-related genes. A total of 1,566 COVID-19 related genes were acquired from Genecards (https://www.genecards.org/). The RNA sequencing data of TCGA-LUAD patients was obtained from the TCGA data portal (https://tcga-data.nci.nih.gov/tcga/).

## Author Contributions

LD, YXi, and YXu: statistical analysis. XC, YC, and XZ: acquisition of data. FC: obtaining funding. All authors contributed to the article and approved the submitted version.

## Conflict of Interest

Author YX was employed by YuceBio Technology Co., Ltd. The remaining authors declare that the research was conducted in the absence of any commercial or financial relationships that could be construed as a potential conflict of interest.

## Publisher's Note

All claims expressed in this article are solely those of the authors and do not necessarily represent those of their affiliated organizations, or those of the publisher, the editors and the reviewers. Any product that may be evaluated in this article, or claim that may be made by its manufacturer, is not guaranteed or endorsed by the publisher.
