# Supplementary material for: The Potential Bioactive Components of Nine TCM Prescriptions Against COVID-19 in Lung Cancer Were Explored Based on Network Pharmacology and Molecular Docking
Source: Front Med (Lausanne). 2022 Jan 20;8:813119. doi: 10.3389/fmed.2021.813119 (PMC8811133; doi:10.3389/fmed.2021.813119)
Supplement: Supplementary file 1 [file Data_Sheet_1.DOCX]

**Figure S1 KEGG and GO enrichment analysis of immune target genes.Figure S2 The Chinese medicine - constituent -immune target regulatory network of each prescription**

**Figure S3** For the degree distribution and eigenvector centrality distribution of 9 networks, A-I are QFPDT、HSZFZ、SDYFZ、FPQXZ、QYLFZ、SRYFZ、HSYFZ、QYLXZ、YDBFZ networks in turn.

**Figure S4** **GO and KEGG analysis for the targets of each prescription**

**Figure S5 A: The expression and distribution of 8 genes were different between cancer and adjacent cancer; B: Univariate prognostic significance forest map of 8 genes; C: Correlation between 8 genes and ACE2 expression.**
