# Supplementary figures and images for: The Potential Bioactive Components of Nine TCM Prescriptions Against COVID-19 in Lung Cancer Were Explored Based on Network Pharmacology and Molecular Docking
Source: Front Med (Lausanne). 2022 Jan 20;8:813119. doi: 10.3389/fmed.2021.813119 (PMC8811133; doi:10.3389/fmed.2021.813119)

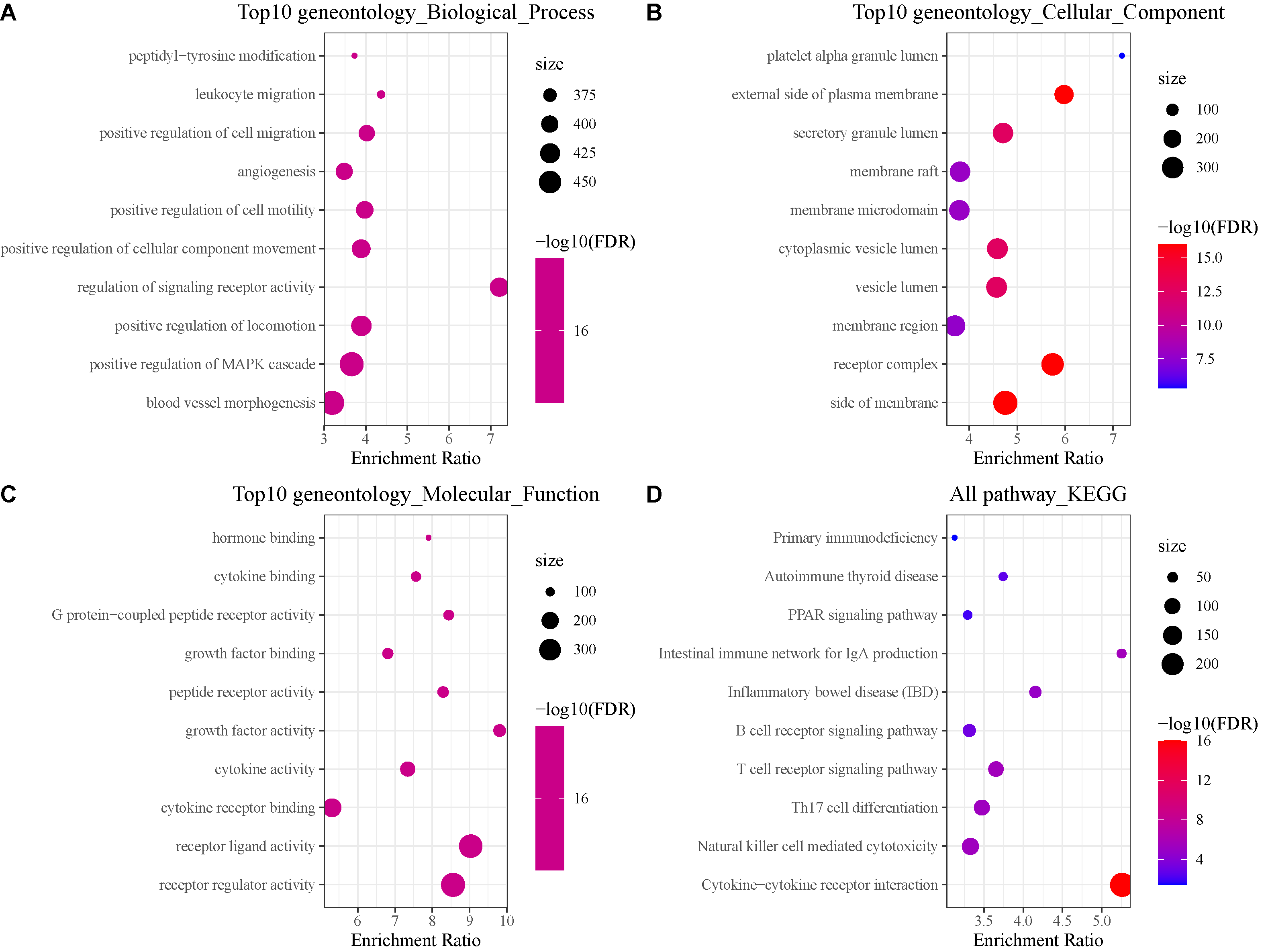

Supplement: Supplementary file 2 [file Image_1.TIF]

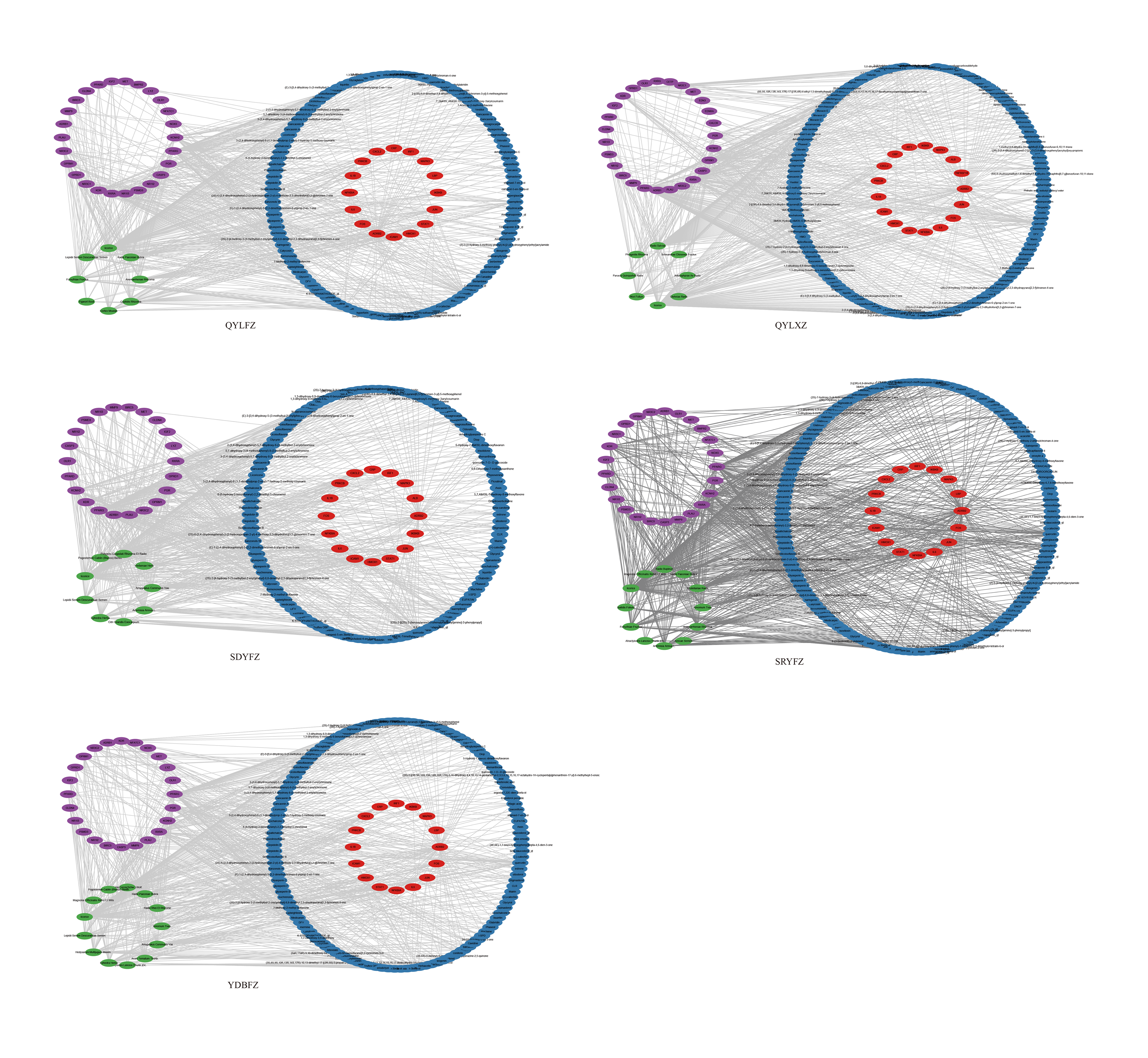

Supplement: Supplementary file 3 [file Image_2.TIFF]

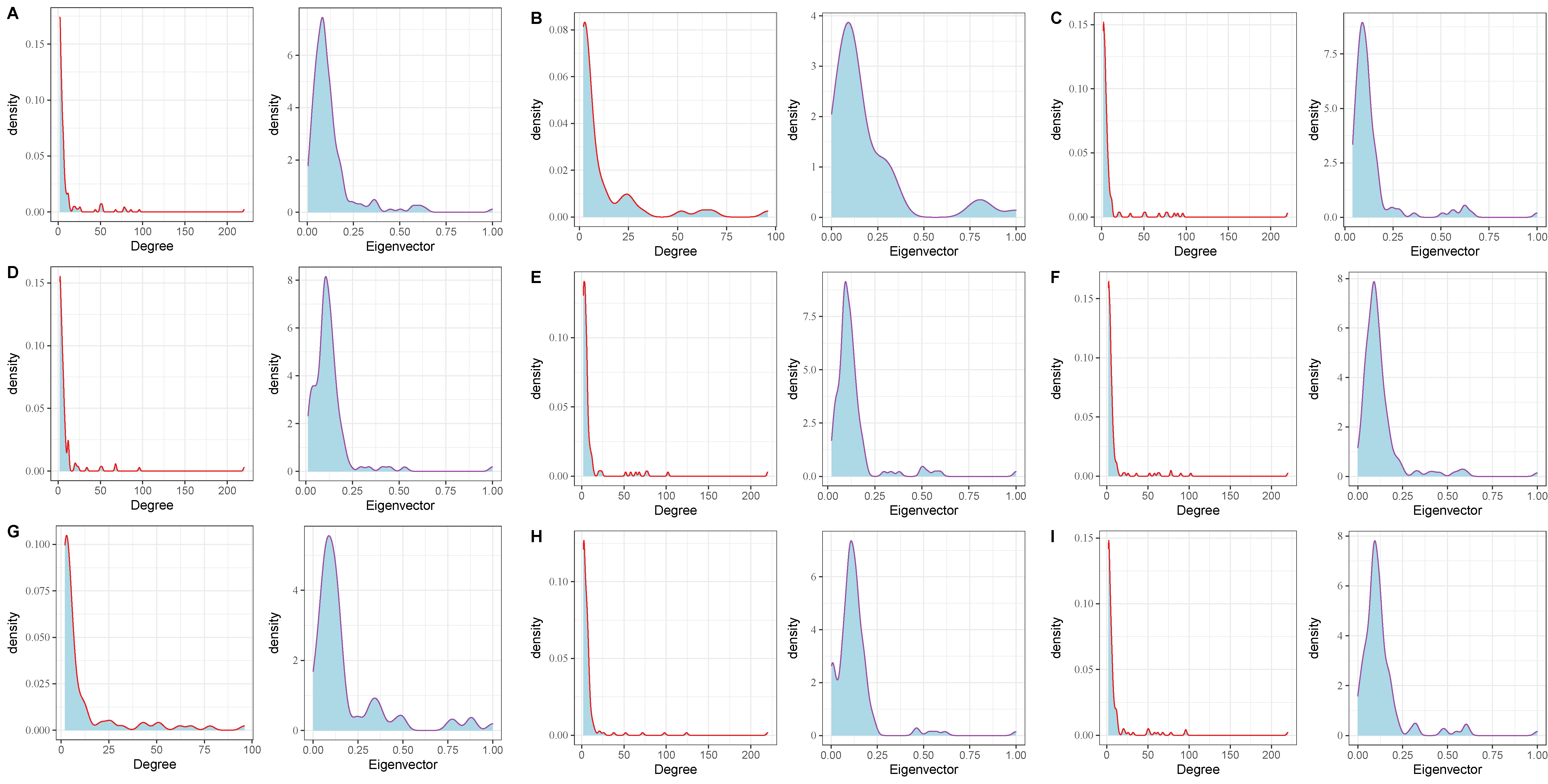

Supplement: Supplementary file 4 [file Image_3.TIF]

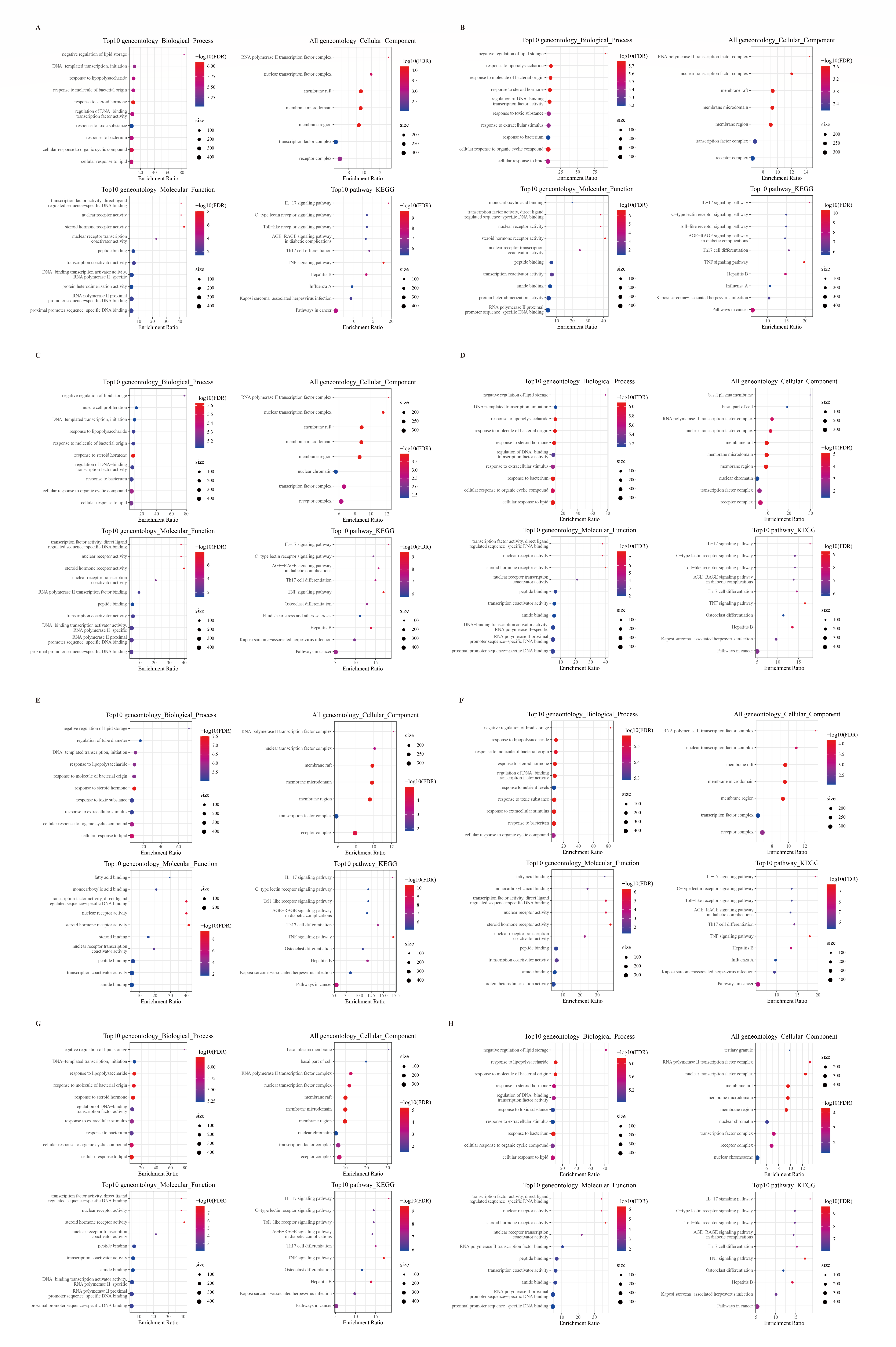

Supplement: Supplementary file 5 [file Image_4.TIFF]

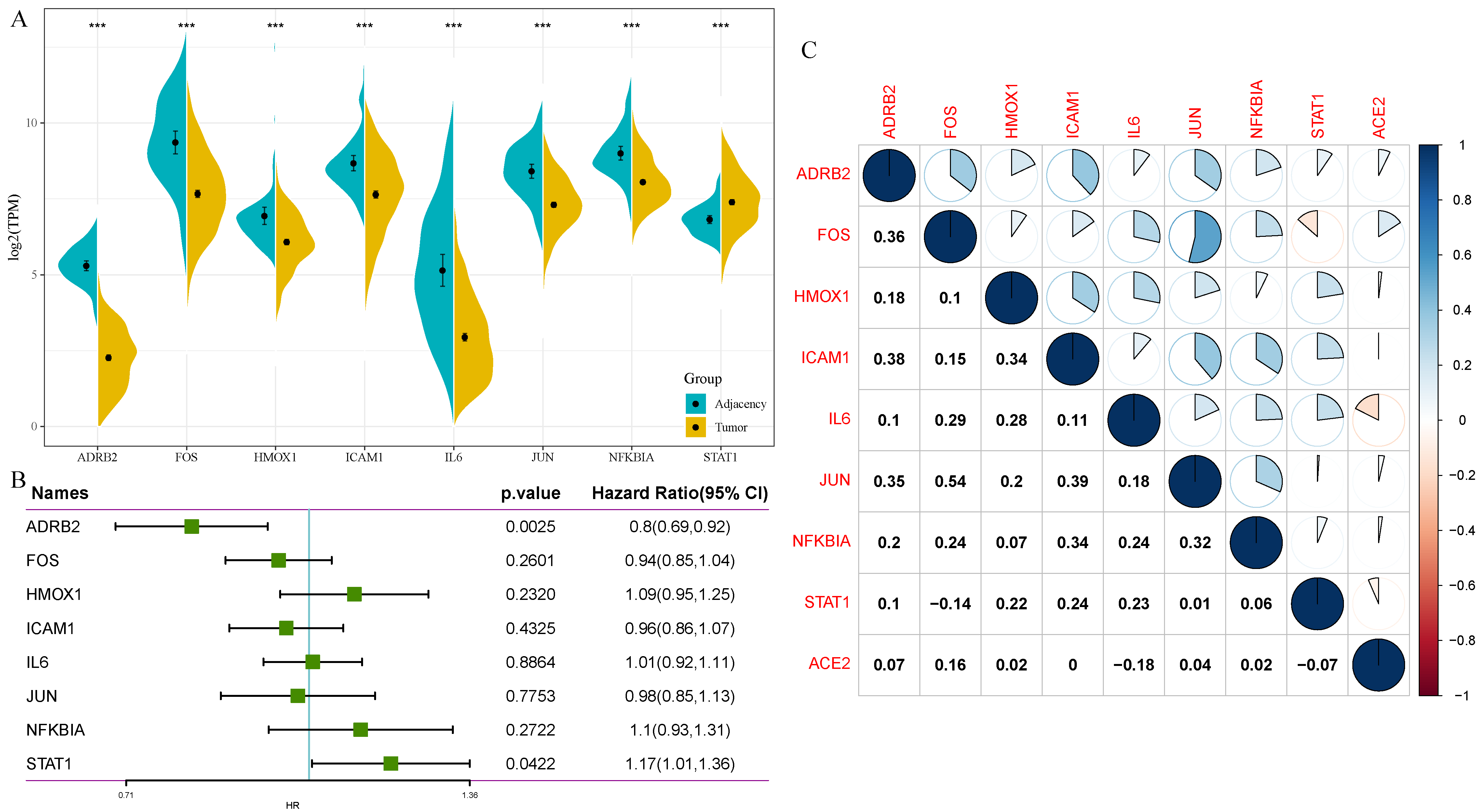

Supplement: Supplementary file 6 [file Image_5.TIF]
